# *SLC6A1* variant pathogenicity, molecular function and phenotype: a genetic and clinical analysis

**DOI:** 10.1093/brain/awad292

**Published:** 2023-08-30

**Authors:** Arthur Stefanski, Eduardo Pérez-Palma, Tobias Brünger, Ludovica Montanucci, Cornelius Gati, Chiara Klöckner, Katrine M Johannesen, Kimberly Goodspeed, Marie Macnee, Alexander T Deng, Ángel Aledo-Serrano, Artem Borovikov, Maina Kava, Arjan M Bouman, M J Hajianpour, Deb K Pal, Marc Engelen, Eveline E O Hagebeuk, Marwan Shinawi, Alexis R Heidlebaugh, Kathryn Oetjens, Trevor L Hoffman, Pasquale Striano, Amanda S Freed, Line Futtrup, Thomas Balslev, Anna Abulí, Leslie Danvoye, Damien Lederer, Tugce Balci, Maryam Nabavi Nouri, Elizabeth Butler, Sarah Drewes, Kalene van Engelen, Katherine B Howell, Jean Khoury, Patrick May, Marena Trinidad, Steven Froelich, Johannes R Lemke, Jacob Tiller, Amber N Freed, Jing-Qiong Kang, Arthur Wuster, Rikke S Møller, Dennis Lal

**Affiliations:** Genomic Medicine Institute and Epilepsy Center, Cleveland Clinic, Cleveland, OH 44195, USA; Universidad del Desarrollo, Centro de Genética y Genómica, Facultad de Medicina Clínica Alemana, Santiago de Chile 7610658, Chile; Cologne Center for Genomics (CCG), Medical Faculty of the University of Cologne, University Hospital of Cologne, Cologne 50931, Germany; Genomic Medicine Institute and Epilepsy Center, Cleveland Clinic, Cleveland, OH 44195, USA; Department of Biological Sciences, Bridge Institute, USC Michelson Center for Convergent Bioscience, University of Southern California, Los Angeles, CA 90089, USA; Institute of Human Genetics, University of Leipzig Medical Center, Leipzig 04103, Germany; Department of Epilepsy Genetics and Personalized Medicine, The Danish Epilepsy Centre, Dianalund 4293, Denmark; Department of Genetics, University Hospital of Copenhagen, Rigshispitalet, Copenhagen 2100, Denmark; Children’s Health, Medical Center, Dallas, TX 75235, USA; Department of Pediatrics, University of Texas Southwestern Medical Center, Dallas, TX 75390, USA; Cologne Center for Genomics (CCG), Medical Faculty of the University of Cologne, University Hospital of Cologne, Cologne 50931, Germany; Clinical Genetics, Guys and St Thomas NHS Trust, London SE19RT, UK; Epilepsy Program, Neurology Department, Hospital Ruber Internacional, Madrid 28034, Spain; Research and Counseling Department, Research Centre for Medical Genetics, Moscow 115478, Russia; Department of Neurology and Metabolic Medicine, Perth Children’s Hospital, Perth 6009, Australia; School of Paediatrics and Child Health, UWA Medical School, University of Western Australia, Perth 6009, Australia; Department of Clinical Genetics, Erasmus MC, University Medical Center, Rotterdam 3015GD, The Netherlands; Department of Pediatrics, Division of Medical Genetics and Genomics, Albany Medical College, Albany Med Health System, Albany, NY 12208, USA; Department of Basic and Clinical Neurosciences, Institute of Psychiatry, Psychology and Neuroscience, King’s College, London SE58AF, UK; Department of Basic and Clinical Neurosciences, King’s College Hospital, London SE59RS, UK; Department of Pediatric Neurology, Amsterdam Public Health, Amsterdam University Medical Center, Amsterdam 1081HV, The Netherlands; Department of Pediatric Neurology, Stichting Epilepsie Instellingen Nederland (SEIN), Heemstede and Zwolle 2103SW, The Netherlands; Division of Genetics and Genomic Medicine, Department of Pediatrics, St.Louis Children’s Hospital, Washington University School of Medicine, St. Louis, MO 63110, USA; Autism and Developmental Medicine Institute, Geisinger, Danville, PA 17837, USA; Autism and Developmental Medicine Institute, Geisinger, Danville, PA 17837, USA; Department of Regional Genetics, Anaheim, Southern California Kaiser Permanente Medical Group, CA 92806, USA; Pediatric Neurology and Muscular Diseases Unit, IRCCS Istituto Giannina Gaslini, Genoa 16147, Italy; Department of Neurosciences, Rehabilitation, Ophthalmology, Genetics, Maternal and Child Health, University of Genoa, Genoa 16132, Italy; Department of Clinical Science, Kaiser Permanente Bernard J. Tyson School of Medicine, Pasadena, CA 91101, USA; Department of Paediatrics, Regional Hospital of Central Jutland, Viborg 8800, Denmark; Department of Paediatrics, Regional Hospital of Central Jutland, Viborg 8800, Denmark; Centre for Educational Development, Aarhus University, Aarhus 8200, Denmark; Department of Clinical and Molecular Genetics and Medicine Genetics Group, VHIR, University Hospital Vall d’Hebron, Barcelona 08035, Spain; Department of Neurology, Université catholique de Louvain, Cliniques universitaires Saint-Luc, Brussels 1200, Belgium; Centre for Human Genetics, Institute for Pathology and Genetics, Gosselies 6041, Belgium; Department of Pediatrics, Division of Medical Genetics, Western University, London, ON N6A3K7, Canada; Medical Genetics Program of Southwestern Ontario, London Health Sciences Centre and Children's Health Research Institute, London, ON N6A5A5, Canada; Department of Paediatrics, Division of Pediatric Neurology, London Health Sciences Centre, London, ON N6A5W9, Canada; GeneDx, Gaithersburg, MD 20877, USA; Department of Medical Genetics, UPMC Children’s Hospital of Pittsburgh, Pittsburgh, PA 15224, USA; Medical Genetics Program of Southwestern Ontario, London Health Sciences Centre, London, ON N6A5W9, Canada; Department of Neurology, Royal Children’s Hospital, Melbourne, VIC 3052, Australia; Department of Pediatrics, University of Melbourne, Melbourne, VIC 3052, Australia; Murdoch Children’s Research Institute, Melbourne, VIC 3052, Australia; Genomic Medicine Institute and Epilepsy Center, Cleveland Clinic, Cleveland, OH 44195, USA; Luxembourg Centre for Systems Biomedicine, University of Luxembourg, Esch-sur-Alzette 4362, Luxembourg; Translational Genomics, BioMarin Pharmaceutical Inc., Novato, CA 94949, USA; Translational Genomics, BioMarin Pharmaceutical Inc., Novato, CA 94949, USA; Institute of Human Genetics, University of Leipzig Medical Center, Leipzig 04103, Germany; Center for Rare Diseases, University of Leipzig Medical Center, Leipzig 04103, Germany; SLC6A1 Connect, Frisco, TX 75034, USA; SLC6A1 Connect, Frisco, TX 75034, USA; Department of Neurology, Vanderbilt University Medical Center, Nashville, TN 37240, USA; Neuroscience Graduate Program, Vanderbilt University, Nashville, TN 37235, USA; Department of Neurology, Vanderbilt Brain Institute, Nashville, TN 37235, USA; Department of Pharmacology, Vanderbilt University, Nashville, TN 37232, USA; Vanderbilt Kennedy Center of Human Development, Nashville, TN 37203, USA; Translational Genomics, BioMarin Pharmaceutical Inc., Novato, CA 94949, USA; Department of Epilepsy Genetics and Personalized Medicine, The Danish Epilepsy Centre, Dianalund 4293, Denmark; Department of Regional Health Research, University of Southern Denmark, Odense 5000, Denmark; Genomic Medicine Institute and Epilepsy Center, Cleveland Clinic, Cleveland, OH 44195, USA; Stanley Center of Psychiatric Research, Broad Institute of Harvard and MIT, Cambridge, MA 02142, USA; Department of Neurology, University of Texas Health Sciences Center at Houston, Houston, TX 77030, USA

**Keywords:** autism, epilepsy, neurodevelopmental disorder, genetics, SLC6A1

## Abstract

Genetic variants in the *SLC6A1* gene can cause a broad phenotypic disease spectrum by altering the protein function. Thus, systematically curated clinically relevant genotype-phenotype associations are needed to understand the disease mechanism and improve therapeutic decision-making.

We aggregated genetic and clinical data from 172 individuals with likely pathogenic/pathogenic (lp/p) *SLC6A1* variants and functional data for 184 variants (14.1% lp/p). Clinical and functional data were available for a subset of 126 individuals. We explored the potential associations of variant positions on the GAT1 3D structure with variant pathogenicity, altered molecular function and phenotype severity using bioinformatic approaches.

The GAT1 transmembrane domains 1, 6 and extracellular loop 4 (EL4) were enriched for patient over population variants. Across functionally tested missense variants (*n* = 156), the spatial proximity from the ligand was associated with loss-of-function in the GAT1 transporter activity. For variants with complete loss of *in vitro* GABA uptake, we found a 4.6-fold enrichment in patients having severe disease versus non-severe disease (*P* = 2.9 × 10^−3^, 95% confidence interval: 1.5–15.3).

In summary, we delineated associations between the 3D structure and variant pathogenicity, variant function and phenotype in *SLC6A1*-related disorders. This knowledge supports biology-informed variant interpretation and research on GAT1 function. All our data can be interactively explored in the SLC6A1 portal (https://slc6a1-portal.broadinstitute.org/).

## Teaser

*SLC6A1* encodes GABA transporter protein type 1 (GAT1). Stefanski *et al*. investigate the relationship between SLC6A1 pathogenic variants, GAT1 structure and function, and clinical phenotype in SLC6A1-related disorders. Biology-informed variant interpretation can improve therapeutic decision-making and guide drug development.

## Introduction

*SLC6A1* encodes for the GABA transporter protein type 1 (GAT1), a membrane protein responsible for GABA neurotransmitter reuptake from the synaptic cleft in inhibitory synapses.^1^*SLC6A1*-related developmental and epileptic encephalopathy (DEE) is an autosomal dominant genetic disorder. Clinical manifestation of *SLC6A1* DEE is characterized by childhood onset seizures and mild-to-severe intellectual disability. Seizure types include absence, myoclonic and atonic. Language impairment and behavioural problems have also been observed.^2-4^ Other frequently observed *SLC6A1*-related phenotypes include autism spectrum disorder (ASD) and motor dysfunction, encompassing stereotypies and ataxia. A fraction of patients have shown intellectual disability or ASD without epilepsy (3%).^2^ Recent GAT1 analyses support complete or partial loss-of-function (LoF) as the primary disease-associated molecular pathology, which disrupts the reuptake of GABA.^5-9^

Despite recent aggregation efforts,^2,5,10,11^ there is a need for systematically curated clinically relevant genotype-phenotype associations to understand the disease mechanism and possibly guide genetic counselling, patient management and, ultimately, treatment. It has been shown that 32 out of the 88 (36.4%) described *SLC6A1* patient variants are located in the helical-transmembrane segments and inter-helical hinges. In contrast, general population variants cluster in the cytoplasmic domain.^2^ An analysis using the GAT1 3D structure may increase the granularity of these preliminary observations and identify clinically relevant variant-to-phenotype or variant-to-function associations. 3D structure analysis has previously been successful in elucidating genotype-phenotype associations in various genes.^12-17^ An investigation into gene variant effects across sodium channelopathies showed clustering of pathogenic missense variants in functional domains.^18-22^ However, due to limited available patient data for most *SLC6A1* variants, meaningful associations have been difficult to establish. Currently, *SLC6A1* variant interpretation is still challenging as, to date, there is no single resource with aggregated and curated data for *SLC6A1*-related disorders. Previous studies have suggested that transmembrane segments are important for protein function.^2,4,5,10,11,23^ However, clear guidance on which segments or subdomains are particularly affected is lacking. A recent study on the molecular mechanism of *SLC6A1* variants, investigating 182 variants, showed that LoF variants are found predominantly around the proteins’ vertical axis.^10,11^ A relationship between transporter activity and literature-based disease association has been recently proposed.^11^ However, statistical confirmation of phenotype and variant location needs yet to be established.

The complexity and heterogeneity of *SLC6A1*-related disorders pose difficulties in disease recognition, diagnosis, prognosis and care. The spatial analysis of genetic variants on 3D protein structures has the potential to identify genotype-phenotype correlations, as has been shown in other related neurodevelopmental disorders.^24-30^ As ‘phenotype’ for the analysis, clinical data of variant carriers or molecular readouts generated for the variant can be used to study the effect of different variants.^30^ However, this type of work requires large datasets from various sources. In our study, we build upon previous data aggregation efforts and bioinformatic methods and present the currently most extensive effort to investigate genotype to phenotype associations for *SLC6A1*-related disorders.

Here, we aggregated the currently largest collection of individuals with *SLC6A1*-related disorders and implemented a 3D-based framework^28^ to evaluate genetic, clinical and functional features. Our study compiled a comprehensive dataset of pathogenic and likely pathogenic *SLC6A1* variants from the literature, ClinVar^31^ and our clinical research network. We also incorporated population variants from gnomAD as controls for comparative analysis (gnomAD, public release 2.1.1). Subsequently, we performed linear sequence and 3D protein structure-based genotype-phenotype analysis using *in vitro* assay and clinical phenotype data to identify structure, to function to phenotype relationships for *SLC6A1*-related disorders. Finally, we deployed all data and analysis tools into the SLC6A1 portal, a joint effort of clinical and basic science investigators in collaboration with advocacy groups, to enhance further analysis, awareness and variant interpretation of *SLC6A1*-related disorders.

## Materials and methods

### Genotype and phenotype data from patients with *SLC6A1*-related disorders

We aggregated published genetic and corresponding phenotype data from *SLC6A1*-related disorder studies.^2,5,6,11^ Investigators provided unpublished genetic and phenotype data (*n* = 51) from the Danish Epilepsy Centre, Filadelfia, Denmark (K.M.J. and R.S.M.). We also included genetic and syndrome-level data from the Epi25 collaborative for large-scale whole exome sequencing in the epilepsy collaborative database.^32^ Epi25 data are limited to genotype and International League Against Epilepsy (ILAE) syndrome categorization. The data for all patient variants (*n* = 172) that were evaluated, curated and harmonized in collaboration with clinical experts, including comprehensive annotations, can be viewed in Supplementary Table 1.

The functional data were aggregated from two recent studies.^5,11^ One study quantified GABA uptake for 182 variants from 15 cohorts, including individuals with epilepsy, developmental disorders and healthy controls. The dataset contains pathogenic and likely pathogenic variants, variants of uncertain significance, variants that had been classified as benign or likely benign, and variants that were unclassified or had conflicting annotations.^11^ Additionally, we included functional readouts of two variants (p.Pro361Thr and p.Leu73Phe) from a recent publication.^5^ Patients or their legal guardians provided signed informed consent according to the Declaration of Helsinki and local IRB requirements.

### Genotype data from public repositories

We retrieved general population *SLC6A1* missense variants (*n* = 158) from gnomAD (public release 2.1.1) in Variant Call Format.^33^ Missense variant annotation was performed with variant effect predictor (VEP),^34^ including information from public repositories.^33^ Pathogenic variation in *SLC6A1*-related disorders is mostly *de novo* and rarely expected to be found in general population repositories such as gnomAD.^2^ Thus, we used general population variants from the gnomAD database as controls. Although most variants are expected to be fully penetrant, we also calculated a gnomAD frequency cut-off for ultra-rare *SLC6A1* disorder variants with incomplete penetrance using the cardiodb allele frequency app.^35^ We accessed pathogenic and likely pathogenic ClinVar^31^ missense variants from the FTP site (https://ftp.ncbi.nlm.nih.gov/pub/clinvar/) (ClinVar, July 2021). We obtained ClinVar variants classified as of uncertain significance (VUS) from the FTP site (https://ftp.ncbi.nlm.nih.gov/pub/clinvar/) (ClinVar, December 2022). All genetic variants were mapped onto the canonical isoform, P30531, as defined by the UniProt database (The UniProt Consortium, 2021).

### Domain-specific analysis: mapping variants onto the 3D protein structure

We obtained the human wild-type GABA transporter type 1 3D structure from the Protein Data Bank (PDB ID: 7SK2).^23^ The variants were mapped onto the structure using PyMOL.^36^ For each residue, we calculated a normalized functional score. First, we annotated the functional scores on the GAT1 protein structure using the bio3d R-package.^37^ Second, we normalized the functional activity by calculating the average functional score reported across all residues located within a 5 Å radius. We define the distance from the ligand as the distance in angstroms (Å) between the variant wild-type residue and the ligand. Since no GABA is bound to the GAT1 7SK2 protein structure, we calculated the minimum distance in angstroms between the variant wild-type residue and tiagabine. This GAT1 inhibitor is bound to the GAT1 structure at the GABA binding site (https://www.rcsb.org/structure/7SK2). We considered all atoms of each protein residue and the tiagabine for the minimum distance calculation.

### Functional data curation

We next aggregated data for 184 electrophysiologically tested variants,^5,11^ for which the average transporter activity has been experimentally measured. Both studies have employed a radiolabelled assay to measure the GABA reuptake activity in HEK293T cells. However, Mermer *et al*.^5^ used scintillation counting to quantify the amount of radiolabelled GABA taken up by the cells, whereas Trinidad *et al*.^11^ used mass spectrometry to create a high-throughput GABA trafficking assay. Despite the methodological variations between the two studies, the deviations from previous per cent wild-type (WT) levels were minimal. Mermer *et al*.^5^ conducted their analysis without using two mass spectrometry detectors (MSMS), employed different cell lines that lacked the CRISPR-Cas9 *SLC6A1*-knockout present in the research by Trinidad *et al*.,^11^ and did not account for variable expression efficiencies using the beta-lactamase (BLA)-reporter, as done by Trinidad *et al*.^11^ Each variants’ transporter activity is reported as a percentage of the wild-type activity.^5,11^ All functionally tested pathogenic variants but one (p.Val342Met) showed an LoF effect with an average wild-type activity <42.8%, relative to wild-type activity. The threshold for LoF (42.8%) has been derived from the observed behaviour of ClinVar variants predicted to be synonymous or classified as benign.^11^ To date, pathogenic or likely pathogenic gain-of-function has not been reported.^11^ We stratified all variants into three activity groups based on their average relative-to-WT GABA uptake activity: (i) 0–10%, nearly complete LoF; (ii) 10–42.8%, low activity; and (iii) >42.8%, wild-type.

### Phenotypic data curation

In collaboration with clinical experts, we summarized and harmonized the cognitive and syndrome level data into six cognitive and 12 epilepsy syndrome categories, respectively [cognitive level: severe developmental delay (DD)/ intellectual disability (ID), moderate DD/ID, mild DD/ID, learning disability, unclassified DD, normal; epilepsy syndrome: childhood absence epilepsy (CAE), DEE, epilepsy with myoclonic-atonic seizures (EMAS), intractable primary generalized epilepsy, generalized epilepsy, genetic generalized epilepsy (GGE), intractable absence epilepsy, Lennox-Gastaut syndrome (LGS), non-acquired focal epilepsy (NAFE), temporal lobe epilepsy (TLE), unclassified epilepsy and no seizures]. The classifications were regrouped for consistency by experienced epileptologists (K.M.J. and K.G.). We opted for a binary categorization for disease severity and activity into (i) severe disease; and (ii) non-severe disease. A clinical diagnosis of one of the following syndromes indicates a severe disease: DEE, EMAS, LGS and intractable absence epilepsy. All the patients diagnosed with one of the four syndromes were considered severe because they impose serious life challenges due to their high seizure burden, often with significant developmental delay and are typically resistant to many seizure medications. There was no normal cognition reported in individuals with EMAS after seizure onset. Individuals with no seizures, a diagnosis of CAE, unclassified epilepsy, generalized epilepsy, GGE and TLE or NAFE were classified as having a non-severe disease. Individuals for which no syndrome level data were available or a binary categorization impossible were classified as ‘Other’. The complete regrouping and reclassification can be found in Supplementary Table 1.

### Portal design

The SLC6A1 portal uses the Shiny R framework from RStudio (https://shiny.rstudio.com/) to build the interactive web portal for compatibility, expendability and portability. The portal is publicly available and hosted at the Broad Institute and was deployed with Google Cloud service using a self-contained Docker image (https://slc6a1-portal.broadinstitute.org/). The portal code is available on GitHub (https://github.com/LalResearchGroup/SLC6A1_Portal). We produced a short educational video with whiteboard animation and used the software *VideoScribe* to increase the accessibility to knowledge about *SLC6A1*-related disorders (VideoScribe 3.9.5, Sparkol 2012: https://www.videoscribe.co/en/download/).

## Results

### Data aggregation and description

We present genotype and phenotype data from the largest cohort of individuals with *SLC6A1*-disorders to date, including 172 individuals (DS-172) with 94 unique variants, three copy number variants (CNVs) and 19 recurring variants found in 75 patients (for details on the cohort, see Supplementary Tables 1 and 3). Our clinical dataset of 172 *SLC6A1* variants contains 51 variants that have not been previously published (Supplementary Tables 1 and 2). The most frequent variant in our cohort is p.Val342Met, which was identified in 11 patients. In addition, we report on *in vitro* GAT1 functional readouts for 184 unique variants (DS-184) from two sources.^5,11^ For 70 variants from 126 individuals (DS-126), clinical and *in vitro* transporter function data were available. For 57 variants from 79 individuals (DS-79), clinical and variant information, including syndrome classification, was available together with functionally tested variants to investigate the relationship between disease severity and function (Supplementary Fig. 1 and Supplementary Table 1). Additionally, we mapped 162 of 195 ClinVar missense VUS onto the GAT1 3D protein structure and observed that those variants are dispersed throughout the protein’s structure (Supplementary Fig. 2).

We obtained general population missense variants from gnomAD (*n* = 158) as controls for our analyses. The majority of gnomAD variants included do not overlap with patient variants and only 11 variants (4%) overlap between our patient dataset (*n* = 172) and gnomAD. To explore these variants further, we calculated the maximum allele frequency (AF) in gnomAD (maximum credible population AF = 6.05 × 10^−5^) and added the results to Supplementary Table 1.^35^ The maximum credible population AF was determined using an estimated disease prevalence of 1/619.6 (161.38/100 000),^38^ an incidence of 2.65 per 100 000 live births^39^ and a disease duration of 60.9 years^40^ (1.65 × 60.9 = 100.485). Because the lifespan of individuals with *SLC6A1*-related disorders is unavailable, we selected a population-based estimation of life-years lost. In our cohort, the largest proportion of cases is attributable to the missense variant p.Val342Met, found in 11 of 172 *SLC6A1* disorder patients. The allelic heterogeneity was therefore estimated as 0.06 (11/172), while the genetic heterogeneity, representing the number of genes associated with the disorder, was set to 1 as our study inclusion criterion is a variant in *SLC6A1*. For the above calculation, we estimated an 80% variant penetrance given that few patients have been reported with an inherited variant. Using these parameters to calculate the maximum credible population frequency, we identified a gnomAD variant cut-off of 6.05 × 10^−5^. None of the 11 patient variants that were also present in gnomAD exceeded the threshold set by the maximum credible allele filter.

### Data sharing through the SLC6A1 portal

**Figure 1:**
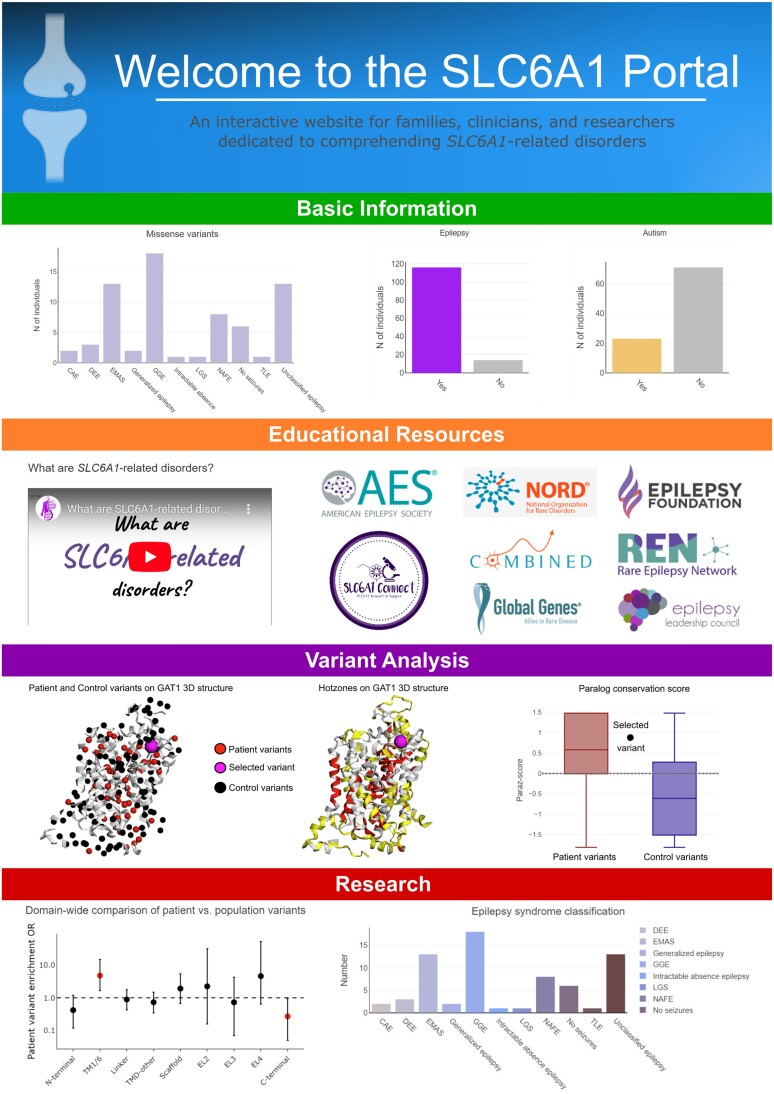
**SLC6A1 portal: user interface and functionality (https://slc6a1-portal.broadinstitute.org/).** Four main menu items hold different functionalities of the SLC6A1 portal. Basic information: key milestones in *SLC6A1* research and summary statistics of the clinical information. Educational resources: resources, links to family advocacy groups and our in-house produced an animated whiteboard explainer video. Variant analysis: clinical significance according to ClinVar and comparative information on the selected variant with other similar variants. Research: visualizations of variant annotations, clinical phenotypes and functional data based on multiple filter options. Efforts to add more data to our online resource are motivated by an increased ability to understand the logic of structure to function to phenotype relations. Furthermore, easy access, the ability to explore the data and educational resources are additional features of our web portal. Given that very few clinicians and caregivers can collect data and perform bioinformatics analyses, the portal enables anyone with access to the internet to explore the data, understand and develop hypotheses.

All the aggregated datasets are integrated to enable scientists to use our rich data source for their research studies and educate providers and families on *SLC6A1*-related disorders. The datasets can be explored in the SLC6A1 portal (https://slc6a1-portal.broadinstitute.org/) (Fig. 1), an interactive and user-friendly web application that combines genetic and clinical data of individuals with *SLC6A1*-related disorders with experimental functional and annotation data on variants. Users can navigate through four sections: (i) basic information; (ii) educational resources; (iii) variant analysis; and (iv) research. By using these data within the portal infrastructure, we enable the exploration of genotype to structure, function and phenotype associations (Supplementary Fig. 4). Here, we present the SLC6A1 portal, which provides access to the largest cohort of patients with *SLC6A1*-related disorders, including their clinical phenotypes and the largest dataset for *in vitro* GAT1 functional readouts. We include a versatile variant entry interface and a visual comparison tool that shows variant location and molecular activity within the GAT1 3D protein structure. Additionally, the portal includes a domain-wide comparison of patient versus population variants and a functional interface for data analysis, including tools to display each variants’ distance from tiagabine versus GABA uptake rate and to identify hot zones on the GAT1 3D structure based on user-selected variant filters. All aggregated data are shared according to the FAIR principles to make it findable, accessible, interoperable and reusable.^41^

### Structure to clinical phenotype relationship

**Figure 2:**
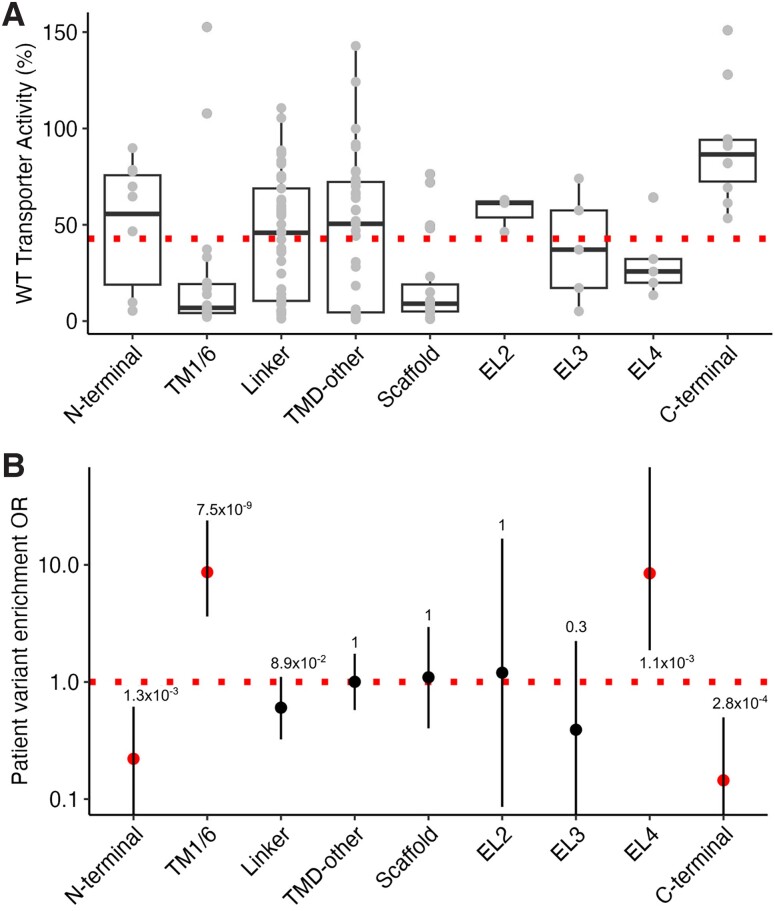
**Domain-wide analysis of patient and population variants.** (**A**) The TM1/6, scaffold and EL4 regions harboured mainly variants with low activity, whereas the N-/C-terminal domain contained mostly variants with wild-type (WT) activity. The dotted line represents the cut-off that separates wild-type activity variants (>42.8% of wild-type activity) from low activity variants (<42.8% of wild-type activity). (**B**) A domain-wide comparison of patient versus population variants shows enrichment of patient variants in TM1/6 and EL4. The N- and C-terminal regions are depleted for patient variants. The dotted line represents a balance between patient and population variants. TM1/6 = transmembrane helix 1/6; TMD-other = transmembrane domain other; EL2/3/4 = extracellular loops 2/3/4; OR = odds ratio.

To elucidate critical regions for transporter function, we retrieved, for each GAT1 residue, its functional domain as classified on the recently published human structure (PDB ID: 7SK2).^23^ We investigated the GABA uptake activity of *SLC6A1* variants by domain and found varying activity levels.^5,11^ Our final dataset contains functional readouts for 156 missense variants (DS-156). It shows that the transmembrane helices 1/6 (TM1/6), scaffold and extracellular loop 4 (EL4) regions harbour 83.3% of variants with low activity (<42.8%) and 16.7% of variants with wild-type activity (>42.8%) (*n* = 40 variants versus *n* = 8 variants with *in vitro* transporter activity <42.8%).^11^ The scaffold in GAT1 comprises helices H3 and H8 and linkers H3-H4 and H8-H9 primarily located in the transmembrane region and serves scaffolding functions.^23,42^ The domain with the lowest average transporter activity of tested variants was scaffold (18.6% average GAT1 activity); the second and third lowest were TM1/6 and EL4, with 23% and 31.1% average activity, respectively. The fourth lowest is extracellular loop 3 (EL3), with 38.2% average activity. The linker, TMD-other and N-terminal domains have an average activity above the threshold of wild-type activity at 42.8% with 44%, 45.9% and 49.4%, respectively, but harbour variants with a wide range of activity levels ranging from nearly complete LoF to wild-type. In contrast, extracellular loop 2 (EL2) and C-terminal regions showed no change in their activity, with 56.9% and 90.5% of average activity, respectively (Fig. 2A). We found no enrichment truncating/frameshift variants across different GAT1 domains. Next, we compared the enrichment of patient versus population variants per region in the *SLC6A1* gene to identify those regions that are predominantly affected by patient variants. For variants that are in the TM1/6 and EL4 regions (*n* = 51), we found an 8.7 and 8.5-fold enrichment of patient versus population variants [TM1/6: 95% confidence interval (CI): 3.6–23.9, *P* = 7.5 × 10^−9^ and EL4: 95% CI: 1.9–78.7, *P* = 1.1 × 10^−3^] (Fig. 2B). Variants in the TM1/6 and EL4 were in agreement with their functional data presented in Fig. 2A. Variants in both the N- and C-terminal regions were depleted for patient variants with an odds ratio of 0.22 for N-terminal (95% CI: 0.06–0.61, *P* = 1.3 × 10^−3^) and 0.14 for C-terminal (95% CI: 0.03–0.5, *P* = 2.8 × 10^−4^). However, only variants in the C-terminal region were concordant with the functional data, as the N-terminal region harboured variants with a wide range of average transporter activity. We observed no enrichment for patient variants in all other regions. The domain-wide analysis of patient versus population variants identified the TM1/6 and EL4 regions as the most essential and the N-/C-terminal as least essential for GABA transport.

### Structure to molecular function relationship

**Figure 3:**
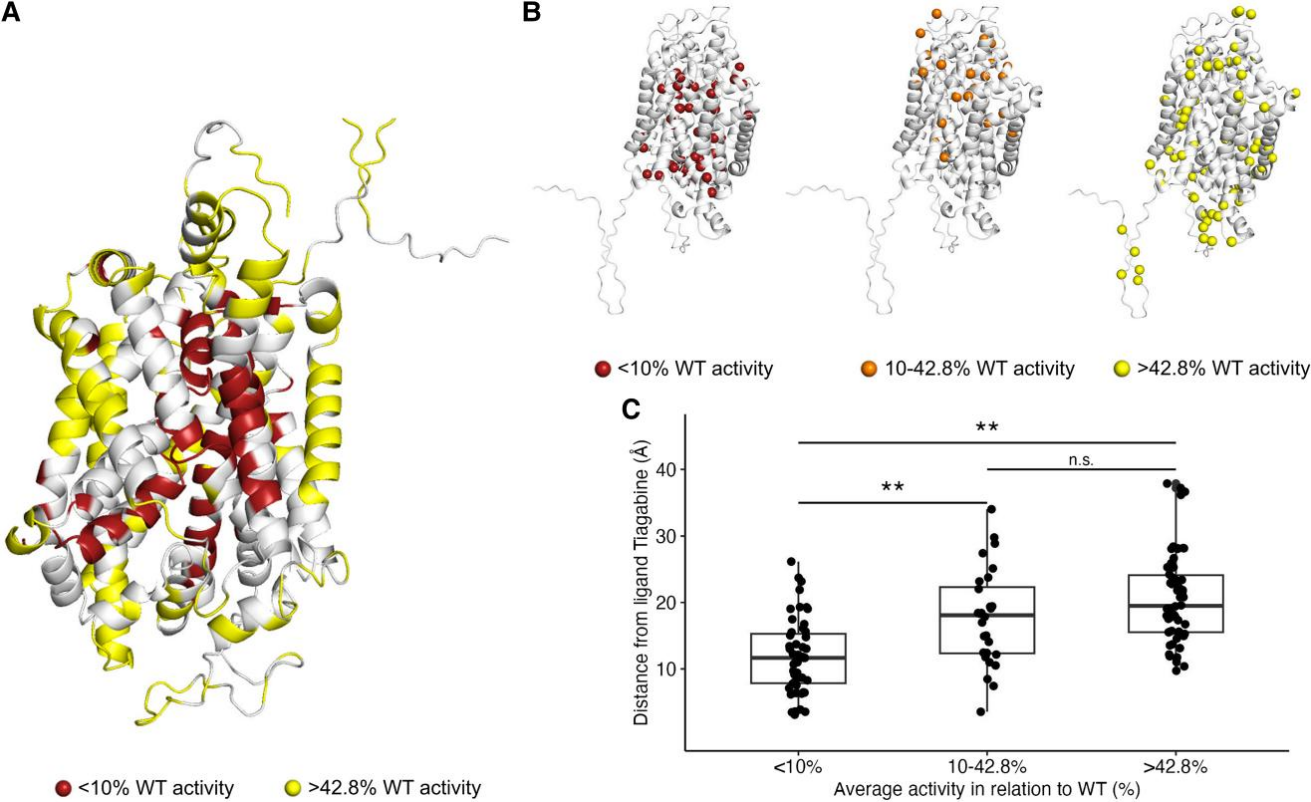
**The spatial relation of *SLC6A1* variants is associated with function and position within the GAT1 protein structure 3D structure (PDB id: 7SK2).** ^23^ (**A**) GAT1 3D structure colour-coded in red regions with nearly complete loss-of-function (LoF) variants (<10% of normalized wild-type activity) and in yellow regions with wild-type activity variants (>42.8% of normalized wild-type activity). (**B**) Side view of the GAT1 3D structure. *SLC6A1* variants were categorized in the same three activity groups [0–10% (*left*), 10–42.8% (*middle*), > 42.8% (*right*)] and mapped onto the GAT1 3D structure. The variants with the lowest and medium average functional activity tend to be closer to the ligand, whereas variants with wild-type activity tend to face outwards. (**C**) Box plot showing the quantification of each variant’s distance (Å) from the ligand by the three activity groups. **Significant after Bonferroni multiple test correction; *Nominally significant; n.s. = not significant; WT = wild-type.

To explore the association between the pathogenicity of a variant and its transporter activity, we localized the missense variants, stratified them into severe LoF (0–10% activity), moderate LoF (10–42.8% activity) and wild-type (>42.8% activity), onto the GAT1 3D structure. Annotated functional scores were normalized over proximal residues across the entire GAT1 3D structure (see the ‘Materials and methods’ section). A visual inspection of the GAT1 3D structure suggests regions that harbour predominantly severe LoF mutations (Fig. 3A and B). A similar analysis on 162 out of 195 ClinVar missense VUS for which a 3D normalized score (for details on the normalized score, see the ‘Materials and methods’ section ) was available did not reveal the same pattern. The variants were dispersed and only 29/162 (17.9%) and 64/162 (39.5%) fall into the complete LoF and moderate LoF group, respectively (Supplementary Figs 2 and 3). We used a spatial distance scoring framework to explore spatial position-to-function relationships that measure each variant’s distance from the ligand.^28^ We calculated the ligands’ distance from each variant in all three activity groups (Fig. 3C) and performed a two-tailed Wilcoxon rank sum test. The <10% wild-type activity group showed the lowest mean ligand distance, and the >42.8% wild-type activity group was the highest (*P* = 2.2 × 10^−10^). Overall, we observed that the higher the average transporter activity, the greater its distance from the ligand in a subanalysis with removed missense variants whose wild-type residue is glycine or proline residues, which are known to be ‘helix breakers’.^43,44^ We found the lowest mean ligand distance for variants in the <10% wild-type activity group and the highest for the >42.8% wild-type group (*P* = 1.1 × 10^−8^) (Supplementary Fig. 5). When considering variants whose wild-type residue was either glycine or proline, an insufficient number of variants remaining impeded a meaningful result (Supplementary Fig. 6). We also investigated the distance from the GABA transporter axis (defined as the distance in angstroms of each variant from the GAT1 axis), from TM1 and from TM6. Similarly, we observed an increasing average transporter activity with increased distance from the transporter axis, TM1 and TM6. Variants in the <10% wild-type activity group showed the lowest mean transporter axis distance, and the >42.8% wild-type activity group showed the highest for the transporter axis, TM1 and TM6. The strongest signal observed was the distance from TM6 (*P* = 8.6 × 10^−9^) (Supplementary material, ‘Methods’ section and Supplementary Figs 7–9).

### Molecular function to clinical phenotype relationship

The clinical spectrum of *SLC6A1*-related disorders is broad. To determine if *in vitro* functional readouts are associated with disease severity, we compared the number of patients with severe versus non-severe disease for variants grouped by their average functional activity in relation to wild-type (0–10%, complete LoF, 10–42.8%, moderate LoF and >42.8%, wild-type). Severe disease is defined by a reported high seizure burden, often with significant developmental delay and refractory seizures resulting in pronounced life challenges and inability to support themselves as prevalent in epilepsy syndromes such as DEE or LGS (see the ‘Materials and methods’ section). For variants with complete loss of *in vitro* GAT1 reuptake, we found a 4.6-fold enrichment of patients with severe versus non-severe disease (*P* = 2.9 × 10^−3^, 95% CI: 1.5–15.3) compared to variants with moderate LoF and wild-type function. Other comparisons were not significant.

## Discussion

We aggregated *SLC6A1* data from various sources to perform joined genetic, protein structure, molecular and clinical data analysis to study genotype-phenotype relationships in *SLC6A1*-related disorders. We show that a reduced distance from the ligand was associated with a greater reduction in transporter activity and that lower GAT1 transporter function is associated with more severe phenotypic *SLC6A1*-related disorder presentations marked by significant life challenges (i.e. intractable epilepsy, moderate-to-severe intellectual disability and ASD). To make all aggregated data available for educational purposes and research projects, we developed the SLC6A1 portal (https://slc6a1-portal.broadinstitute.org/). In addition to data access, we provide tools that allow the evaluation of genetic, clinical and functional features of *SLC6A1*-related disorders on the GAT1 3D structure.

Patient and population variant positions along its protein sequence or 3D protein structure can inform whether a particular variant within a specific region is more likely to cause disease.^45-48^ Previous studies suggested that pathogenic missense variants primarily cluster near the GABA binding pocket, located around the sixth and seventh transmembrane domains of the GAT1 protein.^4,10,11^ However, quantification and validation using a statistical approach were lacking. We demonstrate for the first time that the variants within the transmembrane region of GAT1 are predominantly LoF. Additionally, we observe that missense variants classified as VUS are dispersed throughout the protein’s structure and that 42.6% of those variants fall into the wild-type group (>42.8% wild-type activity) and 17.9% fall into the complete LoF group (<10%), indicating that potentially a subset of VUS might be pathogenic. Future patient variant reports and functional analysis will likely resolve the clinical significance of this subset of VUS. As previously suggested, we could not confirm any cluster of low-activity variants within the seventh transmembrane domain.^4,10^ Further, we refine the association using two orthogonal approaches. First, we investigated domain-wide changes in the transporter activity of tested patient variants. Second, we compared the enrichment of patient versus population variants across all domains and demonstrated that the TM1/6 and EL4 domains harbour variants exclusively with a high decrease in *in vitro* transporter assay activity (<42.8%) compared to wild-type. Both TM1/6 and EL4 were enriched for patient variants. In line with our observation, previous studies on paralogous *SLC6A1–14* genes also pinpoint the TM1/6 region as crucial based on substantial sequence conservation in central regions of the protein structure and the sodium ion binding sites.^49-54^

We show, for the first time, a spatial association of transporter activity and the distance between the variant position and the ligand tiagabine. We could statistically quantify that the distance from the ligand was significantly different for the nearly complete LoF activity group of variants (<10%) compared to the 10–42.8% and >42.8% average activity groups to wild-type groups. Overall, the greater the distance of a variant from the ligand, the closer the transporter activity was to wild-type activity and vice versa. Our results align with previous observations from our team and other research groups that indicated that missense variants with a higher decrease in activity compared to wild-type were predominantly located in the protein’s transmembrane domain and suggested upon visual inspection that LoF variants might be enriched near the transporter axis.^11^ Concordant with the suggestive preliminary data by Trinidad *et al*.,^11^ our 3D variant mapping also shows that variants with very low to low activity, compared to wild-type, tend to be closer to the proteins’ vertical axis. In contrast, variants with wild-type-like activity tend to face outwards, meaning that those variants tend to be consistently on the exterior of the protein structure and might have less interference with the protein’s transporter function. This pattern agrees with GAT1 function as a gateway for GABA, and anything that could obstruct that gateway is potentially impairing its function as a transporter.^11,23,55^ We observed individual variants clustering around the vertical protein axis, causing a decreased channel function through defective transporter function. However, it is important to note that impaired trafficking to the cell surface in cell culture has been demonstrated also to cause channel dysfunction.^5,6,11^ Variants that cause channel dysfunction through impaired trafficking are, however, not captured in our analysis. Also, an assay in human embryonic kidney (HEK) cells is not a model for investigating cellular trafficking. Further trafficking-specific functional evaluation of these variants is required to determine a trafficking defect.

Additionally, it has been shown for related proteins from the SLC6A1–14 transport family that the transmembrane region around transmembrane helices 1 and 6 is a crucial element for a functioning transporter protein.^49-51,56-59^ Although previous studies could show a relation between function and pathogenicity^5,11^ in selected *SLC6A1* variants, no study has yet established an association with disease severity. This study could show now that *in vitro* functional readouts of *SLC6A1* variants were associated with disease severity. We observe an enrichment of LoF variants near the ligand. As this area is essential for GABA transport, disruptions within the transmembrane domain can cause devastating disease, potentially due to complete LoF—instead of partial LoF. Other research groups also identified crucial regions within the transmembrane domain near the GAT1 axis.^2,11,23,54^ We found that variants with low activity compared to wild-type and disease severity were associated with a 4.6-fold enrichment of patients with severe disease versus non-severe disease. Here, severe disease is defined as having major challenges in life, such as any report of refractory seizures, developmental delay or intellectual disability. Children with non-severe phenotypes have fewer challenges, such as no refractory or milder seizures (see the ‘Materials and methods’ section). We could not delineate an association of the ligand distance with the age of seizure onset as this feature was only reported for a small subset of individuals (*n* = 27). This was expected considering previous information from studies on *SLC6A1* that were all limited by small cohort sizes and sparse clinical information.^5,10,11^ Researchers had previously encountered this same challenge with other genes but could overcome these limitations over time with more data.^60^ Examples from the literature show a clear path for exploiting the relationship between variant location and patient phenotype.^60-64^

Our study has several limitations. First, although our *SLC6A1* patient cohort is the largest to date, it is still small, which may prevent findings from being extrapolated. Further, the clinical data have not been ascertained through standardized procedures, instead were *post hoc* curated by clinical experts who ascertained data from many sites. For rare diseases, like *SLC6A1*-related disorders, that only recently have been identified with specific phenotypes, such as specific EEG features, the task of correctly coding phenotypic information in routine care represents a challenge.^65-67^ Cohort size and data standardization both affect statistical analysis power. Notably, the variant position-based analysis performed in this study does not account for trafficking defects that have been shown to contribute to *SLC6A1* deficiency.^5^

Nevertheless, we validated previous suggestive evidence and identified novel genotype-phenotype associations. Data from the current prospective natural history of disease studies^68^ and larger retrospective data aggregations will likely identify additional genotype-phenotype associations and potentially enable risk prediction models.^20,69,70^ Another limitation of this study is the lack of complete genome and environmental data. We noticed that several patients with the same recurrent variant had heterogeneous (non-severe versus severe) expressions of *SLC6A1*-related disorders. For example, the most frequent recurring variant p.Val342Met is classified as CAE for one individual, EMAS for three, and unclassified epilepsy in four individuals with cognition ranging from normal to severe DD/ID (Supplementary Table 1). Owing to the clinical *SLC6A1* heterogeneity, the categorization into non-severe versus severe is limited. This was confirmed in personal discussions with the treating physicians to rule out a coding bias. Future studies should investigate genetic modifiers such as rare variation, the polygenic risk for epilepsy, autism or low intelligence quotient (IQ)^71-74^ since several recent studies showed that genomic background could modify the expression of the disease.^75-78^ Similarly, environmental factors, including drug history, need to be incorporated into statistical models.^75^

In summary, our results show the relationship between each variant’s distance from the ligand and the level of average transporter activity in *SLC6A1*-related disorders. Future functional characterization of variants is needed to investigate the hypothesis presented in this study and to determine whether the association between genetic location and disease severity found in this study can also be found in other clinical phenotypes, such as age at seizure onset. More data need to be aggregated to develop a reliable pathogenicity predictor, as this would be a major step forward in improving the clinical management of patients with *SLC6A1*-related disorders. Our SLC6A1 portal will contribute to this endeavour. Future studies could potentially elucidate the relationship between variant location and treatment response, paving the way for a personalized medicine approach.

## Supplementary Material

awad292_Supplementary_Data

## Data Availability

All relevant data and methods are reported in the article and the Supplementary material.
