# Supplementary material for: SLC6A1 variant pathogenicity, molecular function and phenotype: a genetic and clinical analysis
Source: Brain. 2023 Aug 30;146(12):5198–208. doi: 10.1093/brain/awad292 (PMC10689929; doi:10.1093/brain/awad292)
Supplement: awad292_Supplementary_Data [file awad292_supplementary_data.zip › brain-2022-02416-File006.pdf]

Supplementary Material

Supplementary methods

Mapping variants onto the GAT1 3D protein structure

We define the distance from the transporter axis as the distance in Angstrom (Å) from each variant to the GAT1 axis. We predicted the GAT1 transporter axis using the Mole2.0 web server (https://mole.upol.cz/, v.2.5) and obtained the annotation of those residues that were predicted to be lining the vertical protein axis. We then calculated each protein residue's minimum distance to the GAT1 transporter axis.

The SLC6A1 Portal

The SLC6A1 Portal is structured in four sections, each targeting a different case scenario (Figure 3). The "Variant Analysis" and the "Research" interfaces provide the primary bioinformatics analysis resources. The "Variant Analysis" interface allows the exploration of all variants in the registry for 1) the patient phenotype, 2) functional readouts for 184 variants, 3) ClinVar pathogenicity report, 4) the existence of control variants at a specific position of interest in the general population which is unlikely to be pathogenic for early-onset neurodevelopmental disorders (gnomAD)[26], and 5) similarities/differences of the variant of interest with other variants matching specific criteria, e.g., variants located in the same functional domain. We included pathogenic and likely pathogenic ClinVar variants (n = 34) and population control variants from gnomAD (n = 158) to display a clinical significance assessment and variants from the general population.

The "Research" interface covers several visualizations of subsets of data specified by the user under different tabs:

1. Genotypic information, e.g., variant type, or protein region, and variant location in linear space and on a 3D protein structure
2. The phenotype of the selected variants (e.g., epilepsy syndrome, cognitive level, and others)
3. Known functional *in-vitro* and mouse data

Additionally, the research section contains an interactive interface with the human wild-type (WT) GABA transporter type 1 (GAT1) 3D structure from the Protein Data Bank (PDB ID: 7SK2)[23], including various filter options and the possibility to incorporate reference population missense variants (gnomAD) through a toggle button.[69] All displayed variants can be downloaded for offline analyses.

Detailed information on the data used for the SLC6A1 Portal

We describe 94 unique SLC6A1 variants with three CNVs and 19 recurring variants. The most recurring variant is p.Val342Met, which we observed eleven times. Most patients' variants observed arose de novo (60/78, 77%). Regarding the variant type, we observed 94 missense variants, 21 protein-truncating variants, nine variants in splice sites, three copy number variants, and three small insertions and deletions (Supplementary Table 1). Most of the missense variants likely have a loss-of-function pathogenic mechanism. However, not all are confirmed. In our cohort, epilepsy (117/131, 89%), developmental delay, cognitive impairment (67/79, 85%), and autistic traits (23/94, 25%) were the most common clinical features (Supplementary Table 1). We found a similar number of males and females (54% female), and the mean age of seizure onset was 2.5 years (standard deviation of 1.9 years). Developmental data concerning seizure-onset was available on 67 patients. The most prevalent epilepsy syndrome was early-onset epilepsy with myoclonic atonic seizures (26/103, 25%), followed by genetic generalized epilepsy (19/103, 18%) and non-acquired focal epilepsy (8/103, 8%) (Supplementary Table 1). Previous reports have documented similar proportions (Goodspeed et al., 2020). The mean age of seizure onset in our cohort aligns with previous efforts (2.5 vs. 2.5 years / standard deviation: 1.9 vs. 1.6 years).

Supplementary tables

Supplementary Table 2 – Data Sources

#	Source	Year	Cohort size	Previously published
1	Filadelfia Genetics	2021	52	No
2	Goodspeed et al.	2020	115[a]	Yes
3	Mattison et al.	2018	6	Yes
4	Mermer et al.	2021	22[b]	Yes
5	Trinidad et al.	2022	182	Yes

[a]One individual with a synonymous variant was not considered.
[b]20 out of 22 variants overlap with the Trinidad et al., 2022 study.

Supplementary Table 3 – Cohort description

#	Dataset	# of variants	Description
1	DS-172	172	Patient variants with clinical information from the SLC6A1 Portal
2	DS-184	184	Functionally tested variants
3	DS-126	126	Overlap of DS-172 & DS-184 with clinical information and functionally tested variants
4	DS-79	79	Overlap of DS-172 & DS-184 with clinical information on syndrome level and functionally tested variants
5	DS-156	156	Functionally tested missense variants with distance from ligand available

Abbreviations: DS: Dataset.

Supplementary figures

Supplementary Figure 1

	DS-172	DS-184	DS-156	DS-126	DS-79
DS-172					
DS-184	126				
DS-156	110	156			
DS-126	126	126	119		
DS-79	79	79	74	79	

Supplementary Figure 1 – Overlap between cohorts

Supplementary Figure 2

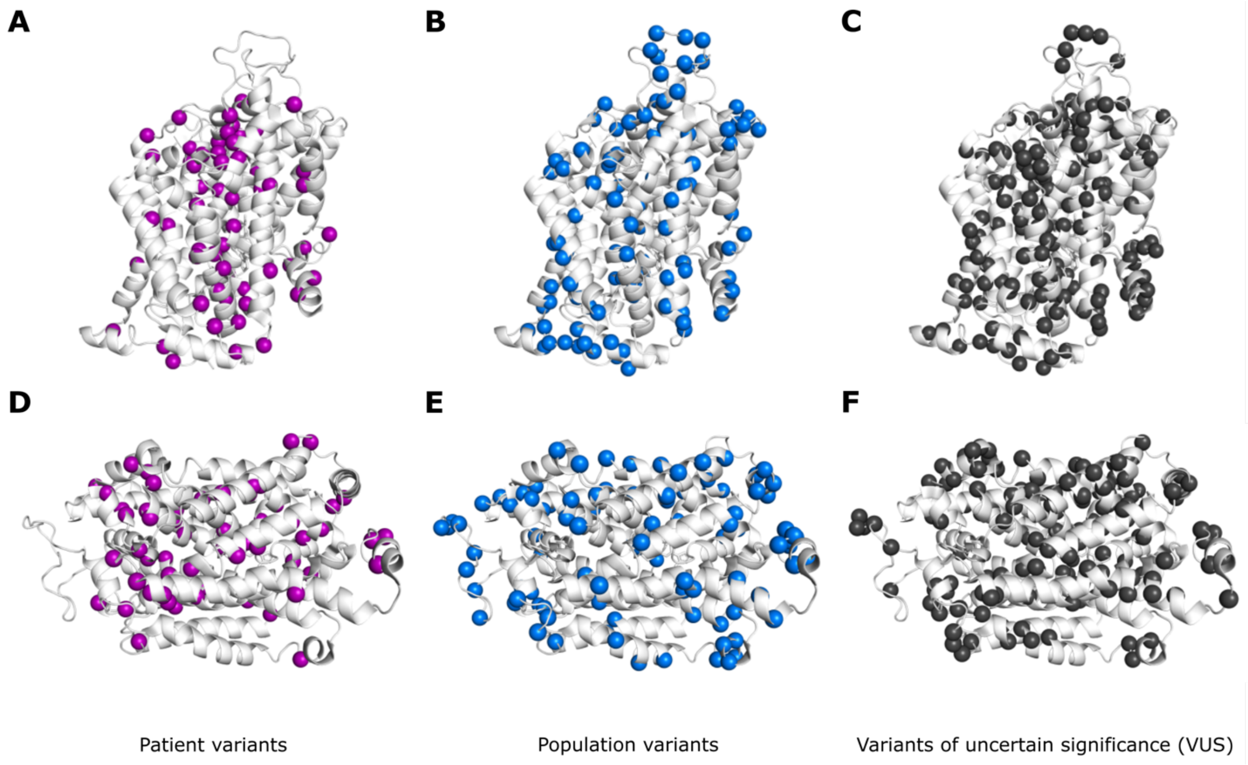

Supplementary Figure 2 – Variant distribution of different study cohorts within the GAT1 3D protein structure. (A, D) Patient variants tend to be closer to the ligand. **(B, E)** Population variants tend to cluster on the periphery of the GAT1 protein. **(C, F)** VUS exhibit high structural heterogeneity and are dispersed throughout the protein's structure. Abbreviations: VUS: Variant of uncertain significance. Abbreviations: VUS: Variant of uncertain significance.

Supplementary Figure 3

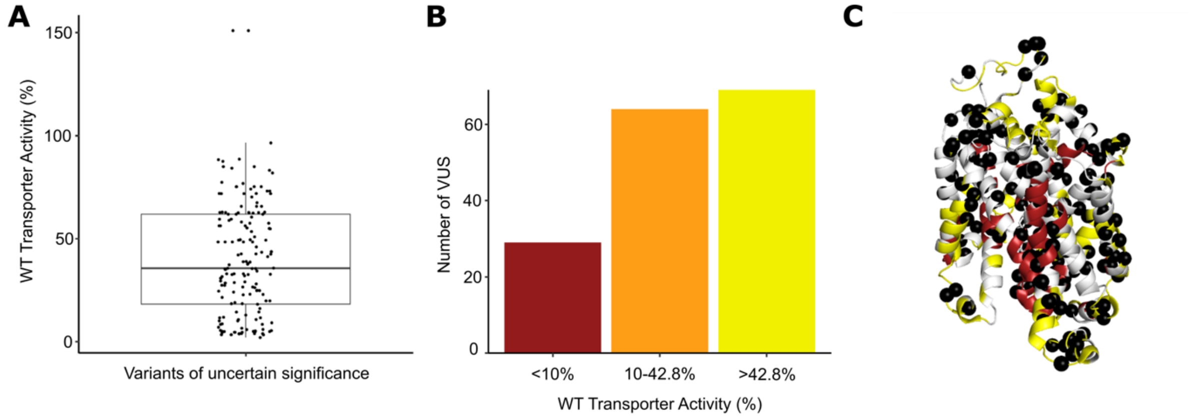

Supplementary Figure 3 – ClinVar missense VUS and their average activity in relation to WT and mapped onto the GAT1 3D protein structure. (A) ClinVar VUS average transporter activity, as measured by in vitro functional assay, in relation to WT. **(B)** Stratification of ClinVar VUS into activity groups, as measured by in vitro functional assay) (0-10% - complete LoF, 10-42.8% - moderate LoF, and >42.8% - WT). **(C)** GAT1 3D structure color-coded in red regions with nearly complete LoF variants (<10% of normalized WT activity) and in yellow regions with WT activity variants (>42.8% of normalized WT activity). ClinVar VUS (black dots) are mapped onto the GAT1 protein structure. Abbreviations: WT: wild-type; VUS: Variant of uncertain significance.

Supplementary Figure 4

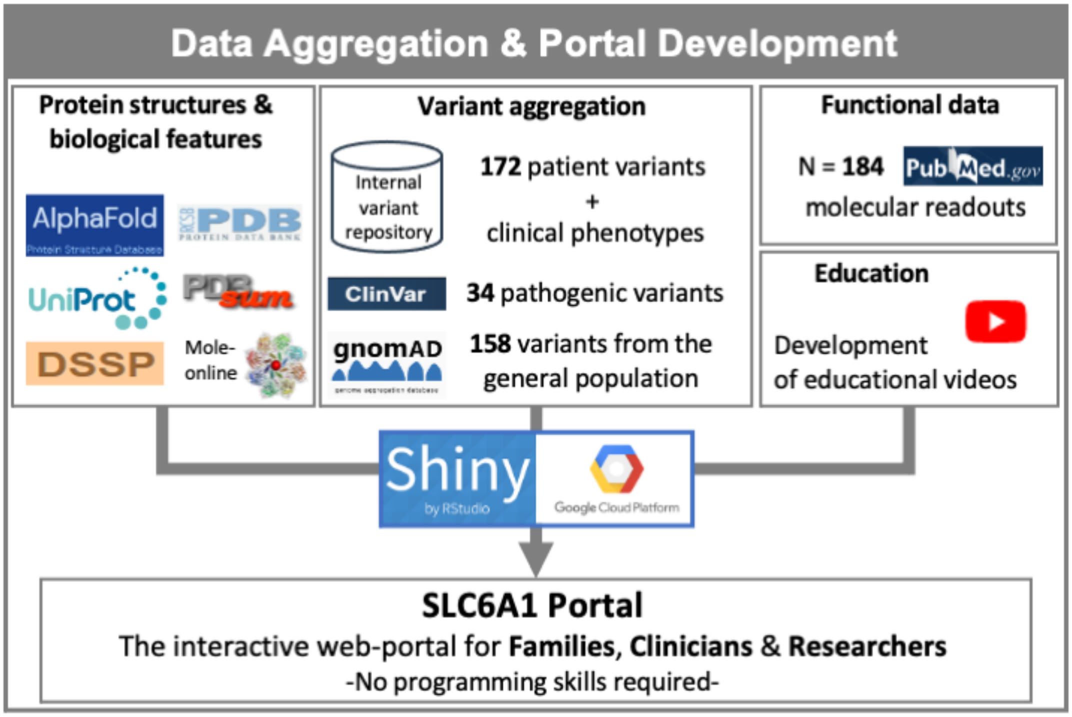

Supplementary Figure 4 – Data Aggregation, cross-referencing, and Portal development show the data access through the SLC6A1 Portal and detail how users interact with the resources. All resources can be accessed through the web-based R Shiny graphical user interphase designed to allow for the download of data, visual interactive variant analysis, protein structure investigations in 2D and 3D, and easy access to educational material.

Supplementary Figure 5

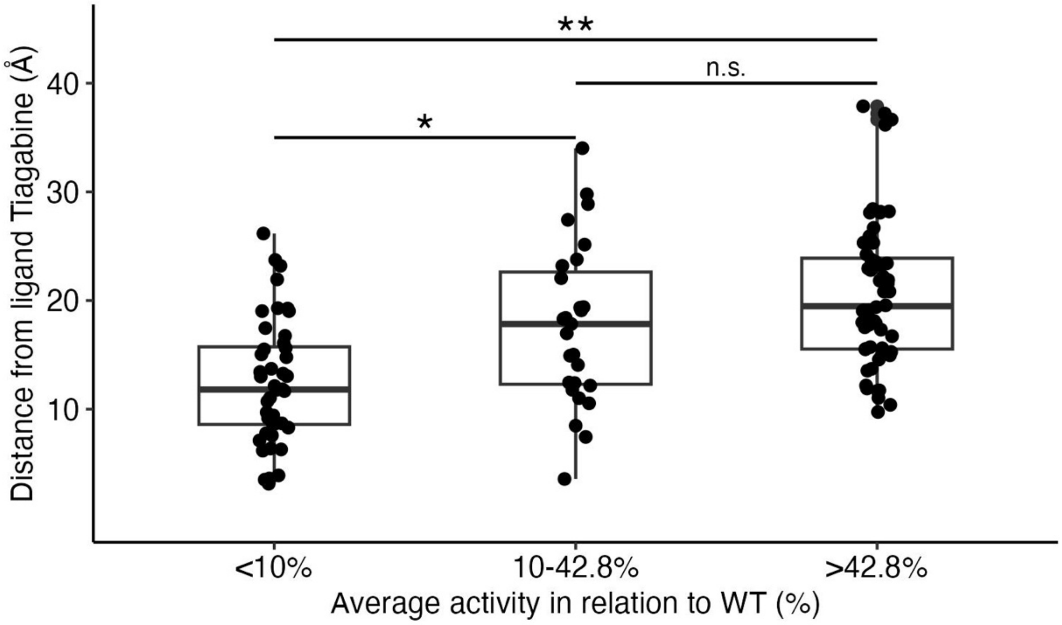

Supplementary Figure 5 – The spatial relation of *SLC6A1* variants is associated with function and position within the GAT1 3D structure (PDB ID: 7SK2).[23] Box plot showing the quantification of each variant's distance (Å) from the ligand by the three activity groups. Missense variants of originally glycine or proline residues are removed from this analysis (n = 144). The <10% WT activity group showed the lowest mean transporter axis distance, and the >42.8% WT activity group was the highest ($P = 1.1e^{-8}$). Abbreviations: **: Significant after Bonferroni multiple test correction; *: Nominally significant; n.s.: not significant; LoF: loss-of-function; WT: wild-type.

Supplementary Figure 6

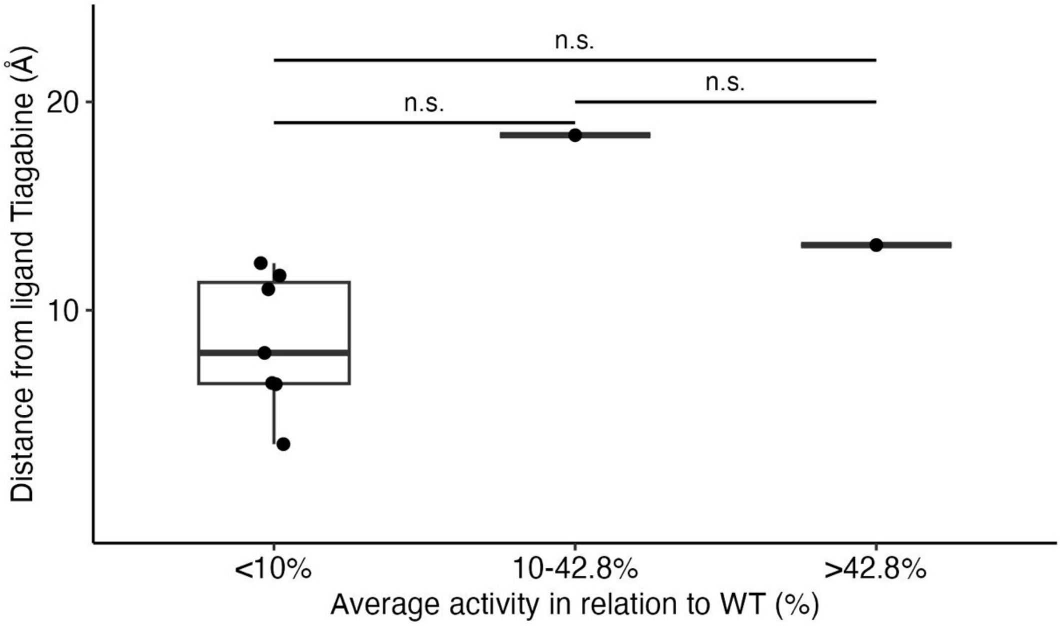

Supplementary Figure 6 – The spatial relation of *SLC6A1* variants is associated with function and position within the GAT1 3D structure (PDB ID: 7SK2).[23] Box plot showing the quantification of each variant's distance (Å) from the ligand by the three activity groups. Only missense variants of originally glycine or proline residues are analyzed (n = 12). The group comparison showed no clear result due to the small number of variants. Abbreviations: **: Significant after Bonferroni multiple test correction; *: Nominally significant; n.s.: not significant; LoF: loss-of-function; WT: wild-type.

Supplementary Figure 7

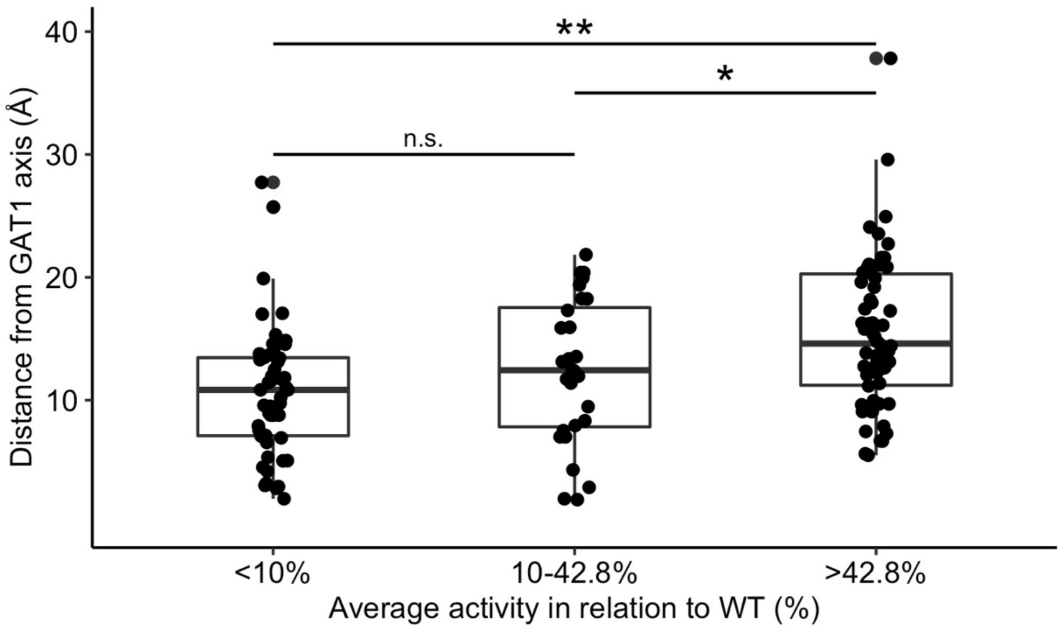

Supplementary Figure 7 – The spatial relation of *SLC6A1* variants is associated with function and position within the GAT1 3D structure (PDB ID: 7SK2).[23] Box plot showing the quantification of each variant's distance (Å) from the transporter axis by the three activity groups. The <10% WT activity group showed the lowest mean transporter axis distance, and the >42.8% WT activity group was the highest ($P = 1.5e^{-5}$). Abbreviations: **: Significant after Bonferroni multiple test correction; *: Nominally significant; n.s.: not significant; LoF: loss-of-function; WT: wild-type.

Supplementary Figure 8

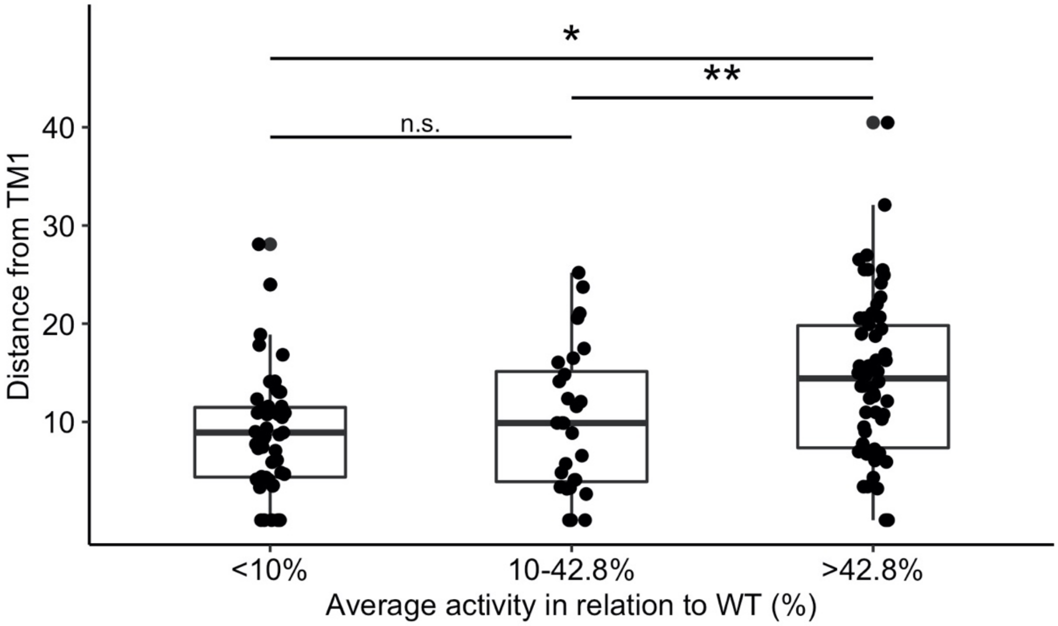

Supplementary Figure 8 – The spatial relation of *SLC6A1* variants is associated with function and position within the GAT1 3D structure (PDB ID: 7SK2).[23] Box plot showing the quantification of each variant's distance (Å) from TM1 by the three activity groups. The <10% WT activity group showed the lowest mean distance from TM1, and the >42.8% WT activity group was the highest (P = 1.1e^{-4}). Abbreviations: **: Significant after Bonferroni multiple test correction; *: Nominally significant; n.s.: not significant; LoF: loss-of-function; WT: wild-type; TM1: Transmembrane domain 1.

Supplementary Figure 9

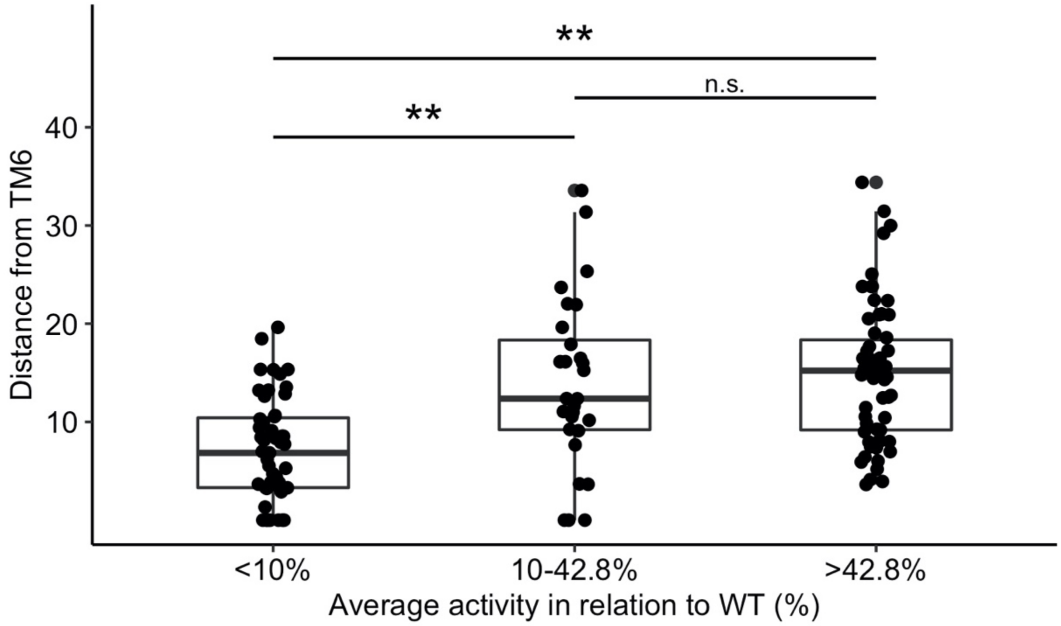

Supplementary Figure 9 – The spatial relation of *SLC6A1* variants is associated with function and position within the GAT1 3D structure (PDB ID: 7SK2).[23] Box plot showing the quantification of each variant's distance (Å) from TM6 by the three activity groups. The <10% WT activity group showed the lowest mean distance from TM6, and the >42.8% WT activity group was the highest (P = 8.6e^{-9}). Abbreviations: **: Significant after Bonferroni multiple test correction; *: Nominally significant; n.s.: not significant; LoF: loss-of-function; WT: wild-type; TM1: Transmembrane domain 6.
